# Ellis-van Creveld Syndrome 2 With Novel Partial Exon 11 Deletion: A Case From Saudi Arabia

**DOI:** 10.7759/cureus.17750

**Published:** 2021-09-06

**Authors:** Nouf Alessa, Mohammed A Mahnashi, Lana Fatehi

**Affiliations:** 1 Pediatrics, King Fahad Central Hospital, Jazan, SAU; 2 Genetics and Metabolism, King Fahad Central Hospital, Jazan, SAU

**Keywords:** ellis-van creveld syndrome, evc, evc2, natal teeth, post-axial polydactyl, dysplasia, saudi arabia, jazan, partial exon 11 deletions

## Abstract

Ellis-van Creveld syndrome (EVC) is a rare genetic disorder characterized by chondral and ectodermal dysplasia. Clinical features may include polydactyly, growth retardation, short ribs, and heart defects. The exact prevalence is still unclear; however, the Amish community in the United States is the most common community to report this rare disease. Until now, only six cases have been reported in Saudi Arabia so far. This is the first case to be reported in the Jazan region. Jazan covers an area of 11,671 km² and has a population of 1,567,547 at the 2017 census. This region has the highest population density with a high consanguinity marriage rate. We present a case of EVC with typical clinical findings, which was confirmed by homozygous mutation in the *EVC2* gene in the region of Jazan, Saudi Arabia. Besides the six cases that were reported from Saudi Arabia, this makes it a total of seven cases. The prenatal findings are considered a good predictor of the disease outcome. More effort is needed in making a national registry of rare disorders to report such cases and provide more awareness among highly consanguinity marriage communities.

## Introduction

Ellis-van Creveld syndrome (EVC) is a rare autosomal recessive disorder. It is characterized by chondro-ectodermal dysplasia. Common clinical findings include polydactyly, growth retardation, short ribs, and heart defects [1-3]. It was first described by Richard Ellis and Simon van Creveld in the 1940s [4]. It is due to defects in two EVC genes, *EVC* and *EVC2* [1,2]. Additional clinical findings that can be variable include dwarfism, dysplastic nails, long, narrow chest, and the presence of natal teeth. In addition, congenital cardiac defects occur in 60% of the affected individuals, with atrial septal defect (ASD) the most common defect [1]. However, EVC is sporadic in the general population with a prevalence of 1 in 60,000 to 200,000 newborns globally [5].

Prevalence in Saudi Arabia is unknown as only six cases have been reported in the country [3,6-9]. We report the first case from Jazan region, Saudi Arabia. Jazan is the second smallest region that stretches around 300 km (190 miles) along the southern Red Sea coast, covering approximately 11,671 km², and has 1,567,547 at the 2017 census. It has a high population density in the Kingdom with a high consanguinity marriage rate [10]. We report a case of EVC with a novel variant making this the seventh reported case in Saudi Arabia.

## Case presentation

**Figure 1:**
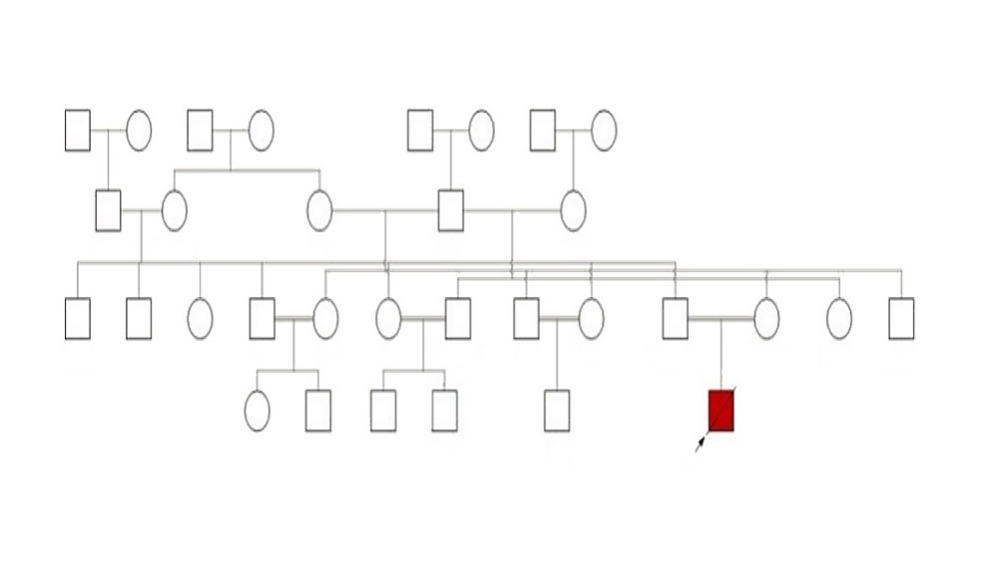
Family pedigree.

**Figure 2:**
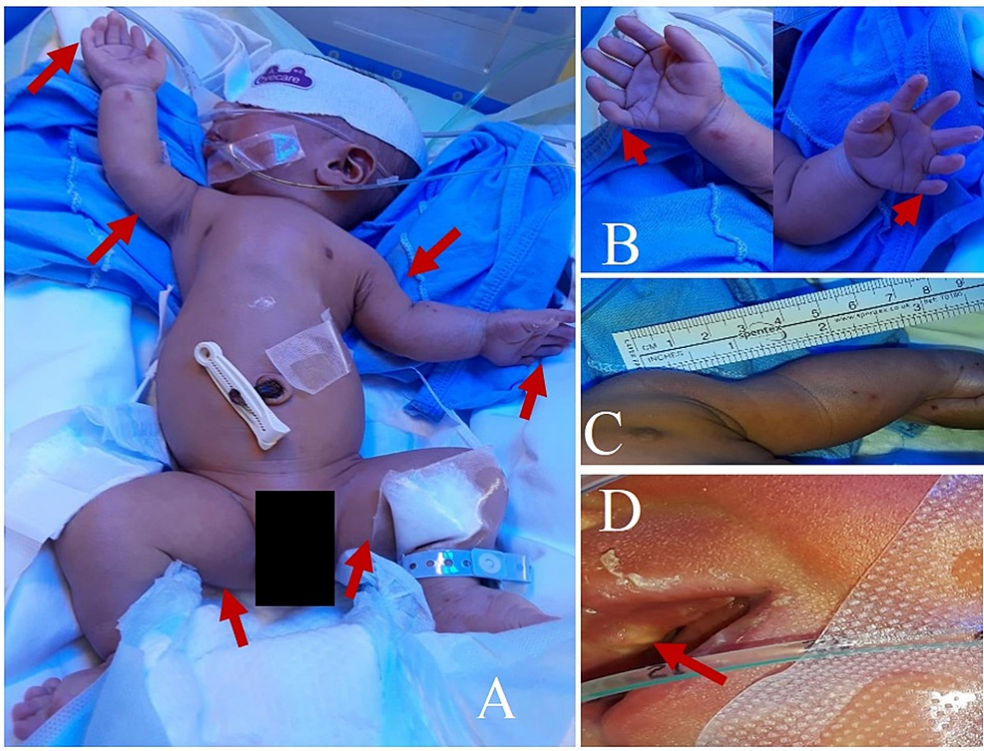
Clinical findings of the baby: A) elongated short thorax with rhizomelic and mesomelic limb shortening. B) Bilateral polydactyly. C) Rhizomelic and mesomelic limb shortening. D) Natal tooth.

We report a full-term male infant that was delivered by cesarean section with strenuous labor due to intrauterine growth restriction and striking dysmorphic features. His parents are first cousins (Figure 1), and he is the first offspring with no history of previous maternal miscarriages. There is no family history of genetic or neurological disorders. Antenatal ultrasound at 34 weeks of pregnancy revealed polyhydramnios and short femur length and humerus length (HL) for gestational age. Measurements included biparietal diameter 83.7 mm equal to 33 weeks, femur length 41.9 mm equal to 23 weeks, HL 35.7 mm equal to 22 weeks and three days, and abdominal circumference (AC) 272.9 mm equal to 31 weeks and three days (Figure 2). He was referred to our hospital as a case of a dysmorphic fetus with a status of high-risk pregnancy center. 

He did not require active resuscitation at birth, with an APGAR score of 7, 9, and 9 at 1, 3, and 5 minutes, respectively. His weight was 2150 grams, length was 41 cm, and head circumference was 33 cm which was below the third percentile. He had a broad forehead, low-set ear, prominent philtrum, two palpable maxillary teeth, high-arched palate, short ribs with a long narrow chest, short limbs, and bilateral postaxial polydactyly in both upper limbs (Figure 2). An echocardiogram revealed a moderate ASD type II, left-right (L-R) shunt, large ASD premium L-R shunt, common aortic valve, dilated right atrium and right ventricle, and small patent ductus arteriosus with L-R shunt.

**Figure 3:**
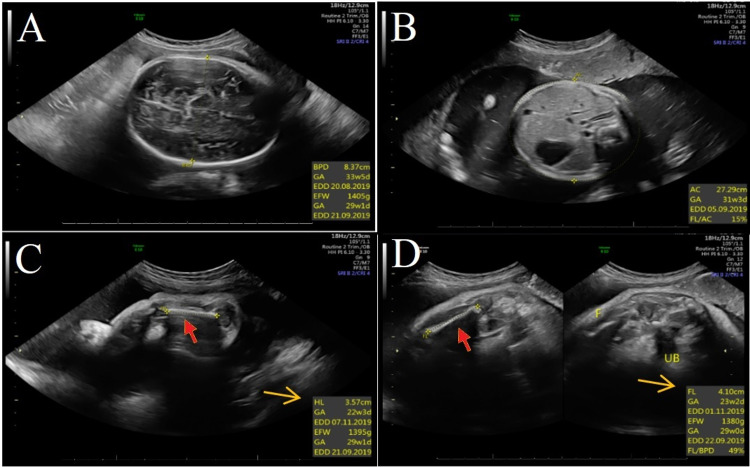
Fetal ultrasound: A) HC; B) AC; C) HL; D) FL. AC: abdominal circumference; HL: humerus length; BPD: biparietal diameter; EDD: estimated date of delivery; EFW: estimated fetal weight; F: fetus; FL: femur length; FL/AC: femur length to abdominal circumference ratio; GA: gestational age; HC: head circumference; UB: urinary bladder.

**Figure 4:**
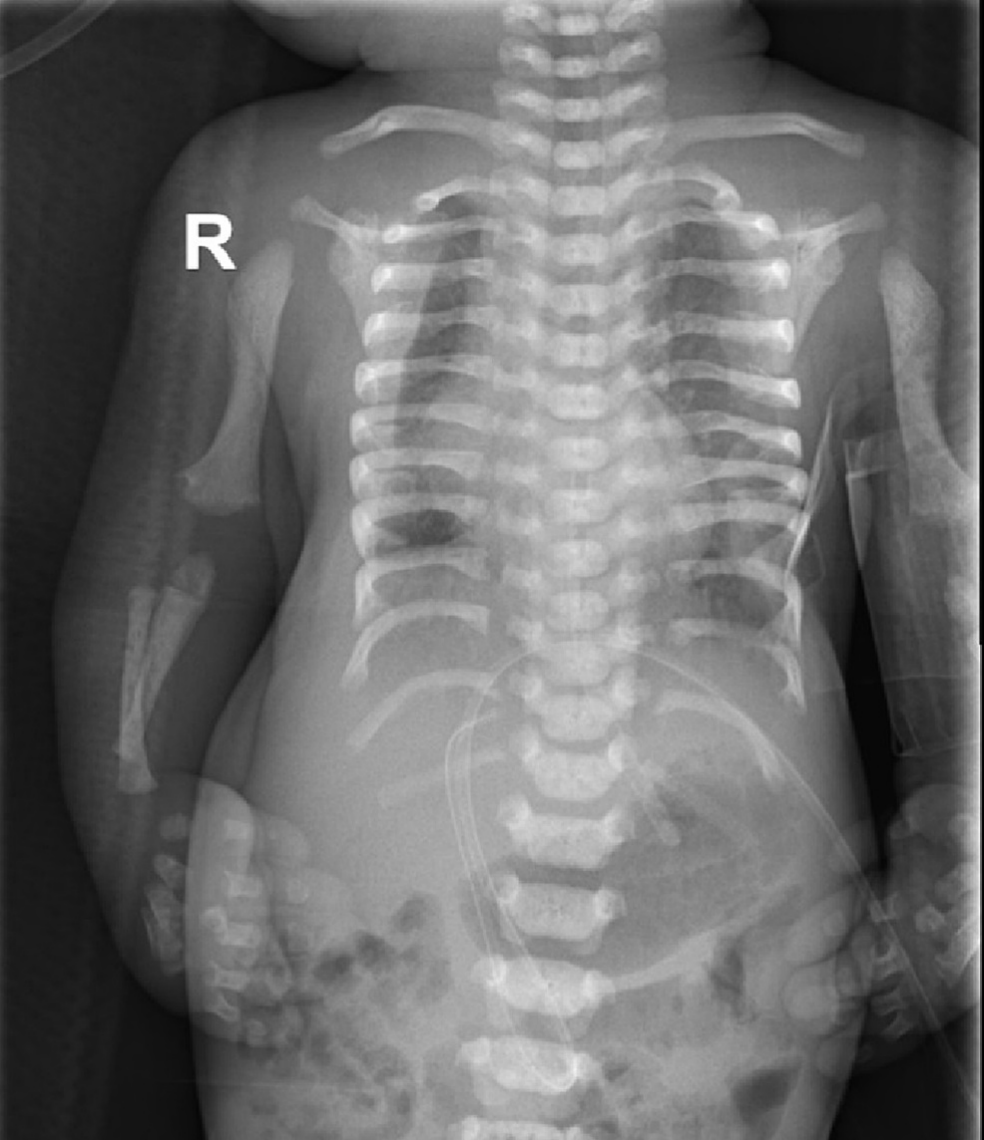
Radiological appearance of the cone-like-shaped epiphysis with short ribs. R: right.

The babygram imaging showed a cone-like-shaped phalangeal epiphysis, short humerus, and thick short ribs (Figures 3, 4). A next-generation sequencing assay for the dysplasia panel including *EVC* and *EVC2* genes were requested in Baylor genetic lab, covering the coding sequence and flanking regions of the genes. The test revealed an exonic, homozygous, likely pathogenic deletion encompassing part of exon 11 of the *EVC2* (NM_147127.4) [11]. The absence of polymerase chain reaction (PCR) amplification of the region confirms this finding. In addition, parents were found to be heterozygous by DNA sequencing for this variant. Unfortunately, other members of the family were not screened for the mutation. After birth, the baby was admitted to the neonatal intensive care unit (NICU) due to respiratory distress and a congenital heart defect. Initially, he required ventilation through nasal cannula. After two weeks, he was started on a continuous positive airway pressure. Unfortunately, due to his complex heart defects, he deteriorated and required intubation and ventilator machine support, then he died. 

## Discussion

EVC is a rare underdiagnosed autosomal recessive chondroectodermal dysplasia syndrome in the Saudi population but is rarely reported with unknown prevalence rate in the country [3,6-8]. Prenatal sonography features were reported previously as a good predictor of EVC diagnosis prenatally [12,13]. Characteristic features postnatally could diagnose EVC, especially in the presence of extra ulnar-side digit (post-axial polydactyly), congenital heart defects, disproportionate limb-to-trunk ratio, natal teeth, and supportive radiological imaging with the appearance of cone-like-shaped epiphysis especially in metacarpals [1-4]. In our patient, prenatal defects were seen prenatally, making the prognosis a bit poor. Furthermore, the patient required immediate NICU admission for respiratory distress and congenital heart defects.

*EVC2* gene encodes a protein that functions in bone formation and skeletal development, but the exact function is unknown [14]. It is located on chromosome 4 with 22 exons. Exon 11 comprises sequences 1471-1710. A deletion of 2 bp was previously reported to cause the EVC (ClinVar) [11]. In comparison, our patient reported a large deletion from the typical EVC reported. With typical clinical features described above, PCR confirmation, and the presence of homozygosity of this variant, we confirm that this variant may be the cause of the patient phenotype. This variant has never been associated with EVC or described in the database or the literature [15]. In the United Arab Emirates (UAE), Ali et al. studied EVC among four families. Common clinical signs among the four families were oral manifestations and nail dysplasia [16]. This was different than our case, as our patient had numerous clinical signs along with heart anomalies. The same study in UAE also found a novel mutation among two families in the depletion of intron 13 of *EVC2* gene while the other two showed nucleotide deletion (c.981delG; p.K327fs) in exon 8 of *EVC2* gene [16]. In Egypt, the EVC was reported by Shawky et al. in 2010 [17]. A total of six patients in their report had similar oral manifestations including the fusion of the upper lip to the maxillary gingival margin and the absence of mucobuccal fold or the sulcus anteriorly [17]. Pectus carinatum was also a distinguishable feature of the patients with narrowing of the chest that led to respiratory distress among them [17].

Consanguineous marriages in Middle East and the Arab countries is still a common practice leading to the birth of many autosomal recessive diseases [16]. However, prenatal genetic testing and carrier screening have shown to provide an effective method in tackling common diseases in Saudi Arabia. However, there are still some genetic syndromes that have been described recently for the first time in Saudi Arabia due to consanguineous marriages such as Woodhouse-Sakati syndrome, spondyloepimetaphyseal dysplasia, and transaldolase deficiency [18]. The incidence of EVC in the Middle East is increasingly reported and a necessary national screening program is recommended to find out more about this syndrome in Saudi Arabia, especially in the southern region where consanguineous marriages are still high.

Up to date, less than 10 cases of EVC have been reported in the Kingdom of Saudi Arabia with none being attributed to Jazan region. However, as part of a vast project to establish a registry of rare genetic disorders, we opted to report those cases to make a good reference in the future.

## Conclusions

In our report, we present the cardinal features needed for the clinical diagnosis of EVC. Utilization of prenatal ultrasound to emphasize the ability of prenatal imaging to detect this disorder and predict the outcome of the disease can be helpful. Genetic analysis in confirming the diagnosis of this rare disorder is crucial and more awareness of these rare genetic disorders' existence is necessary. Creating a registry of rare genetic disorders to initiate preventive measures to decrease autosomal recessive diseases in highly consanguineous communities is required in the future.
